# Regional differences

**DOI:** 10.7554/eLife.30249

**Published:** 2017-08-09

**Authors:** Zhou Yu, Ting Chen

**Affiliations:** 1 Peking University-Tsinghua University-National Institute of Biological Sciences, Joint Graduate Program, School of Life SciencesTsinghua UniversityBeijingChina; 2 National Institute of Biological SciencesBeijingChina

**Keywords:** hair follicle, skin, pattern formation, tissue regeneration, Mouse

## Abstract

The skin is a complex landscape containing regions in which hair follicles exhibit different types of behavior.

**Related research article** Wang Q, Oh JW, Lee HL, Dhar A, Peng T, Ramos R, Guerrero-Juarez CF, Wang X, Zhao R, Cao X, Le J, Fuentes MA, Jocoy SC, Rossi AR, Vu B, Pham K, Wang X, Mali NM, Park JM, Choi JH, Lee H, Legrand JMD, Kandyba E, Kim JC, Kim M, Foley J, Yu Z, Kobielak K, Andersen B, Khosrotehrani K, Nie Q, Plikus MV. 2017. A multi-scale model for hair follicles reveals heterogeneous domains driving rapid spatiotemporal hair growth patterning. *eLife* **6**:e22772. doi: 10.7554/eLife.22772

The regeneration of tissue is a tightly controlled process which ensures that adult stem cells generate an appropriate number of daughter cells in order to maintain healthy tissue in the face of daily wear and tear. Tissue regeneration lasts throughout an animal’s lifetime, but when it goes wrong the end results can include chronic wounds, premature aging and cancer (Fuchs and Chen, 2013). Skin tissue has a fast turnover rate, and many of the fundamental principles that govern tissue regeneration have been discovered in experiments on hair follicle stem cells in mice (Fuchs, 2016).

A hair follicle cycles through three phases: growth, regression and resting. Growth is powered by stem cells being activated to proliferate and generate daughter cells, and the extent to which this happens depends on the relative levels of activating signals (such as WNT) and inhibitory signals (such as BMP; Plikus and Chuong, 2014). However, we know relatively little about the mechanisms that control the levels of these signaling molecules in the first place. Now, in eLife, Qing Nie and Maksim Plikus of the University of California, Irvine and colleagues – including Qixuan Wang as first author – report new insights into these mechanisms based on a combination of simulations and experiments (Wang et al., 2017).

Wang et al. – who are based at Irvine and various institutes in the United States, Korea, China, Australia and Poland – started by using simulations of a simple model to show that certain features of the hair cycle in mice were not observed in the model if it was assumed that all the hair follicles were identical. This indicated that the hair follicles in the different regions of the skin expressed different levels of signal molecules, and that the diffusion of, say, WNT from regions with high levels of WNT influenced the behavior of neighboring regions that had lower levels of WNT to begin with. When the model was adjusted so that dorsal skin hair follicles went through the cycles slower than ventral skin hair follicles, the simulations produced hair cycle patterns similar to those seen in real mice, including waves of hair cycles spreading from ventral skin to dorsal skin.

To test whether or not there exist multiple regions of skin with distinct hair cycles, Wang et al. used a reporter line to detect the level of WNT activity in the skin of live mice in real time. This revealed that hair follicles in ventral chin skin have a shorter growth phase and a faster turnover rate than hair follicles in dorsal skin. To understand the molecular mechanism behind this difference, the researchers discovered that dorsal skin in its resting phase has enriched BMP ligands, depleted BMP antagonists and decreased WNT ligands compared to ventral chin skin. Experiments on transgenic mice confirmed that hair cycles were changed in regions in which BMP or WNT signaling had been perturbed: moreover, perturbing these signals also altered the waves of hair cycles that spread from ventral skin to dorsal skin.

The simulations also predicted that hair follicles would be in an extended resting phase when inhibitor levels are extremely high. Wang et al. observed such behavior in ear skin: the hair follicles in ear skin exist in a dormant resting phase for an extended period of time, with limited or no response to methods that would normally lead to hair growth. The researchers then discovered that the cartilage/muscle complex that is only found in the ear expresses high levels of BMP ligands and multiple WNT antagonists, and went on to show that dampening the BMP signal or increasing the WNT signal can partially activate the growth of hair follicles in the ear.

These experiments confirm that the BMP and WNT pathways control the regional pattern of hair regeneration that is observed, and that these pathways also mediate the interaction among these different regions. This is consistent with the well-known role of BMP/WNT signaling in the skin (Kandyba et al., 2013), but we do not know what gives rise to the intrinsically different levels of BMP and WNT found in the various regions.

Why is it important to understand regional tissue regeneration? Conventional studies often focus on a single hair follicle or a population of hair follicles within one region. These reductionist approaches were able to reveal some of the core principles governing tissue regeneration, but they also missed out on a whole dimension of regulation. Most tissues are composed of heterogeneous cell populations (Donati and Watt, 2015): different regions of skin, for example, differ in hair follicle length, epidermis thickness and pigmentation patterns, all of which serve unique functions (Chang, 2009; Chuong et al., 2013).

Likewise, such regional behavior is evident in many human diseases: for instance, non-segmental vitiligo involves the loss of skin melanocytes from patches of skin on both the left and right sides of the body (Taïeb and Picardo, 2009). These region-specific features suggest that tissue regeneration is regulated by some sort of 'molecular area code'. The next challenge is to work out how this area code works.
